# Acoustic Intensity Causes Perceived Changes in Arousal Levels in Music: An Experimental Investigation

**DOI:** 10.1371/journal.pone.0018591

**Published:** 2011-04-20

**Authors:** Roger T. Dean, Freya Bailes, Emery Schubert

**Affiliations:** 1 MARCS Auditory Laboratories, University of Western Sydney, Milperra, New South Wales, Australia; 2 Empirical Musicology Group, The University of New South Wales, Sydney, New South Wales, Australia; University of Regensburg, Germany

## Abstract

Listener perceptions of changes in the arousal expressed by classical music have been found to correlate with changes in sound intensity/loudness over time. This study manipulated the intensity profiles of different pieces of music in order to test the causal nature of this relationship. Listeners (*N* = 38) continuously rated their perceptions of the arousal expressed by each piece. An extract from Dvorak's *Slavonic Dance Opus 46 No 1* was used to create a variant in which the direction of change in intensity was inverted, while other features were retained. Even though it was only intensity that was inverted, perceived arousal was also inverted. The original intensity profile was also superimposed on three new pieces of music. The time variation in the perceived arousal of all pieces was similar to their intensity profile. Time series analyses revealed that intensity variation was a major influence on the arousal perception in all pieces, in spite of their stylistic diversity.

## Introduction

A mounting body of scientific research confirms the intuitions of many that music can be emotionally expressive [1]–[12]. The literature suggests that in addition to extramusical associations, and expectations arising from culturally familiar musical structures such as harmonic relationships between notes [12], listeners find music emotionally arousing. Arousal is one of the two dimensions in Russell's circumplex model of emotion [13], which has been shown to be widely applicable to listeners' perceptions of music [1], [3], [4], [9]. In common with most authors, we here study perceptions of musical expression rather than an induced arousal response (such as physiological arousal). Expressed arousal is conceptualised along a scale from active to passive, where, for example, ‘angry’ and ‘sleepy’ relate to more active and more passive respectively. This scale can address the energetic and tension components of arousal [14].

Listeners' perceptions of arousal in music seem to be influenced by variations in the basic acoustic property of sound intensity, with its perceptual counterpart of loudness [4], [5], [11]. Bradley & Lang [15] argue that appetitive and defensive systems underpin the expression of emotion through sound. Increases in intensity might well evoke an aversive response, signalling the approach of danger [16]. Temporal profiles of loudness correlate with the temporal profiles of emotional arousal levels that listeners perceive while listening to music [3]. To test whether this relationship is causal, we altered the intensity profiles (on which loudness largely depends [17]) of several pieces without perturbing other musical features: a Dvorak Slavonic Dance previously studied by Schubert [3] was chosen as one of these pieces. If a given piece presented in two versions differing only in intensity profiles generates a perceived arousal profile varying strongly with the intensity profile, this provides direct evidence of a causal relationship specific to intensity. Such an experiment was undertaken here with the Dvorak Slavonic Dance. If arousal profiles were similar across very different pieces displaying the same intensity profile, this would also support this causality. This was achieved with several stylistically diverse new compositions. We support the causal hypothesis here in both respects.

## Materials and Methods

### Methods

#### Ethics Statement

Written informed consent was obtained from all participants, and the study was approved by the Human Research Ethics Committee of the University of New South Wales (Approval No 09 2 006).

The measured intensity profile of part of Dvorak's *Slavonic Dance Opus 46 No 1* [3] was used to create a new version of the piece with logarithmic intensities inverted with respect to median dB SPL (increases become decreases and vice versa – sound file available from the authors on request). Three extracts from compositions by the first author were also studied: two minimal process music computer-piano pieces [18], one completely tonal, one largely atonal; and an electroacoustic piece comprising temporal waves of filtered noise [19].

### Materials

The Dvorak *Slavonic Dance No. 1 in C Major, Opus 46* was from start to 2′18″, while the whole piece lasts 3′52″ in the recording by the Slovak Philharmonic Orchestra, conducted by Zdenek Kosler (Naxos CD 8.550008-09). The Dean tonal (Audio S1) and atonal (Audio S2) extracts were from *Mutase* (2008), and comprised two strands, with a repetitive isochronic (5+5+3 eighth notes, each occupying 180 ms) melodic pattern together with progressive probabilistic variation in individual pitches, in each case in keeping with the tonal or atonal nature. The filtered noise piece was from *soundAffects* [19], an audiovisual performance- and web-piece (Audio S3). The diverse set of extracts was chosen to exemplify, besides variations in intensity, rhythmically active tonal music (Dvorak, *Mutase* tonal), rhythmically active atonal music (*Mutase* atonal), and timbrally rich music (Dvorak, *soundAffects*). There was also strong timbral contrast between the pieces: Dvorak being orchestral (multi-instrument), *Mutase* being realised on a piano (single-instrument), and *soundAffects* using complex noise textures. The pieces also encompass aspects of both the 19^th^ and 21^st^ centuries of Western music composition.

#### Participants

38 students (13 female) aged 19–26 (mean 21 yr) undertook the study. Participants had a median Ollen Musical Sophistication Index [20] of 226 (range 17–956). They all reported normal hearing.

#### Procedure

##### Sound intensity measures

Praat v5 was used for intensity analyses [21] and to manipulate intensity profiles, using ‘intensity tiers’, with minimal concomitant change, such as virtually unchanged spectral flatness profiles. Only the Dvorak showed strong instrumental attack envelopes, and these were slightly perturbed by the intensity transformation as judged by trained listeners.

##### Perceived arousal measures

Participants listened once to all five stimuli over Sennheiser HD280 headphones, rating the perceived arousal of each through time. Stimulus presentation order was randomized, with the constraint that the two versions of the Dvorak should not be presented in immediate succession. Participants were tested individually, in an isolated space. A modified version of the Schubert 2D-emotion space [3], [21] was installed on a Macintosh MacBook. This programme incorporates a training phase presenting participants with detailed instructions on-screen about the arousal scale, followed by exercises in which participants rate the perceived arousal of practice stimuli, with feedback provided. Having satisfied a given accuracy criterion in rating the perceived arousal of verbal stimuli (e.g. the words ‘angry’, ‘sleepy’), participants preceded to the main listening task. Listeners continuously rated their perception of the music's arousal levels (for the distinction between perceived and induced emotion, see [22]) by moving a mouse [21]. They initiated each of the five trials by placing a cursor in a central box on the computer screen. Each trial lasted for the duration of the stimulus, i.e. 2′18″.

The perceived arousal dimension (i.e. how passive or how active the music seems) ranged from −100 to +100. Data were sampled every 250 ms, and subsequently averaged across all participants at each sampled time point (*N* = 557 time points) in order to produce an arousal time series.

## Results

**Figure 1:**
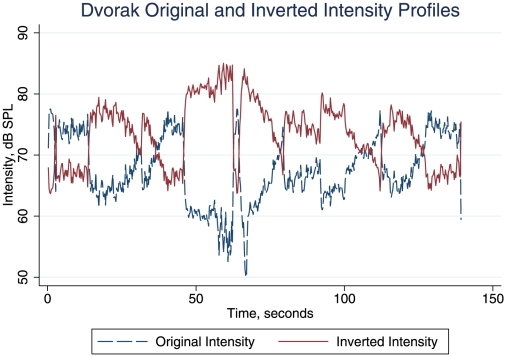
Original and Inverted intensity profiles of the Dvorak *Slavonic Dance Opus 46 No 1.* The dotted blue line represents the intensity in decibels (Sound Pressure Level) as a function of time in seconds of the original recording of the piece. The solid red line shows the intensity profile inverted with respect to median decibels (Sound Pressure Level) through time.

**Figure 2:**
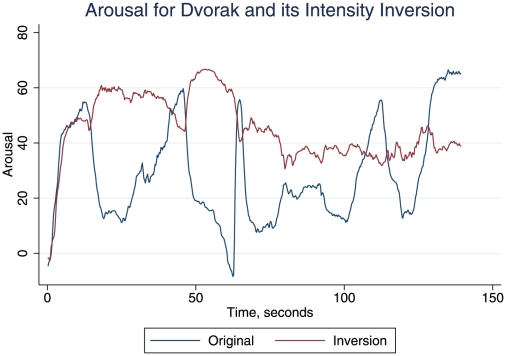
Mean arousal profiles for the Dvorak in its Original and Inverted forms. Perceived arousal through time was measured on a scale from −100 (very passive) to +100 (very active), and averaged across participants at each time point to produce an aggregate arousal response per piece. Arousal ratings for the original form of the piece are indicated in blue, with the perceived arousal of the inverted intensity form of the Dvorak marked in red.

Figure 1 shows the original and inverted intensity patterns. The three newly created piece profiles were extremely similar to the original. Figure 2 shows the arousal profiles for the Dvorak original and its inverted-intensity versions. It is clear that the arousal profile is also inverted when the intensity profile is inverted. Procrustes distances (*d*: where 0 is superimposable and 1 is maximum separation) were measured in order to quantify the distances between the various time series data points studied here (see Gower [23] for a discussion of Procrustes transformations, with examples from many scientific fields). We used unrestricted transformations (which give the minimal distance estimate), and standardised the variables. As Table 1 shows, after the exchange of increase and decrease in the inverted version, the Procrustes distance between the two arousal profiles was small (0.006), consistent with the mirroring effect of inversion of intensity. The *d* values in Table 1 confirm the close relation of arousal profiles to intensity profiles both in the original and its intensity inversion.

**Table 1:** Procrustes values (*d*) between the Dvorak Original arousal time series and other stimulus arousal and Dvorak intensity series.

				Dvorak		
	Tonal *Mutase* Arousal	Atonal *Mutase* Arousal	*soundAffects* Arousal	Inverted Arousal	Original Intensity	Inverted Intensity
Dvorak Original Arousal	0.0001	0.0002	0.0003	0.0006	0.0003	0.0004

**Figure 3:**
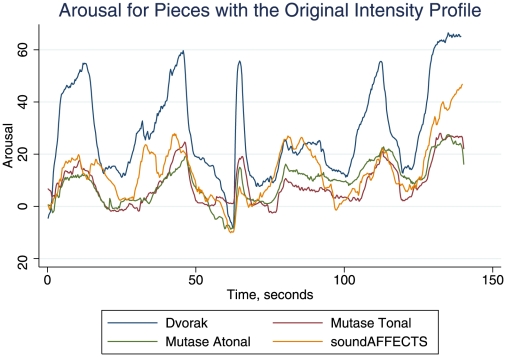
Perceived arousal of the stimuli that feature the Original intensity profile. Mean arousal profiles for the four pieces studied, each bearing the original intensity profile of the Dvorak. Perceived arousal through time was measured on a scale from −100 (very passive) to +100 (very active), and averaged across participants at each time point to produce an aggregate arousal response per piece.

Given this indication that the intensity profile of the Dvorak strongly influenced the perceived arousal profile, we next investigated whether the original profile would similarly influence perceived arousal in diverse unrelated pieces. Figure 3 shows mean arousal ratings for the four pieces sharing the original intensity profile. The arousal profiles were remarkably similar, and all peaks/troughs corresponded to the intensity profile. Arousal perceived in the original Dvorak showed larger peaks where the largest crescendi occur, and it had a larger coefficient of variation (CV) than the others : 0.14 for the Dvorak original vs. 0.07 for both the tonal and atonal pieces. (For determining arousal coefficients of variation, where CV = SD/M, 100 was added to each mean time series value to make them all positive; determining CV of the modified series then permits comparisons between different pieces.) The arousal CV difference may reflect familiarity with the Dvorak genre, and associated perceptual fluency [1]. Yet the CV was only 0.08 for the ‘inverted’ arousal profile, possibly reflecting the introduction of incongruence between intensity and structural features of the music. It may also be relevant that in the inverted version, decrescendi occupy more time than crescendi, and vice versa for the original [24]. Procrustes distances between the arousal curves, which only share the original intensity profile, are shown in Table 1. These small *d* values confirm the similarity of the four temporal profiles of perceived arousal.

**Figure 4:**
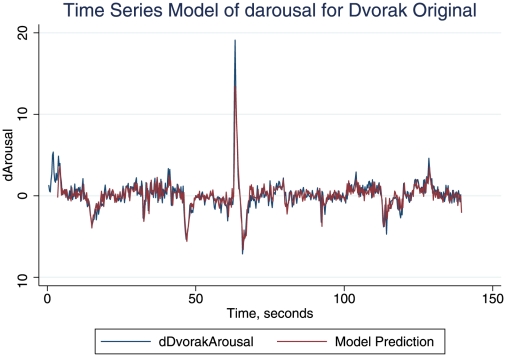
Perceived and modelled arousal in the Original Dvorak composition. Time series analysis (ARIMAX) prediction of the arousal profile of the Dvorak (original form), compared with the measured arousal. The model is statistically highly significant (*p* = .0000). Arousal ratings are differenced (dArousal).

The distances for the arousal/intensity Dvorak relationships were also small (Table 1). Thus the Procrustes distances between input and output response profiles were in all cases small and similar. However, Procrustes calculations disregard the fact that time series showed autocorrelation: so we undertook elaborate time series analyses [25] and confirmed the large predictive power of the intensity series for the perceived arousal. The analyses were done in Stata 10. Relationships between the arousal and intensity time series were assessed with stationarized series, in each case achieved by taking the first difference series (termed dintensity and darousal respectively). Vector autoregression (VAR) was used to test for Granger causality, really an index of correlation: there was highly significant Granger causality (*p*<.01) of dintensity upon darousal in each case. ARIMAX (autoregressive integrated moving average analysis with an exogenous variable, intensity) was undertaken, and in both VAR and ARIMAX highly significant (*p*<.001) models with white noise residuals free of autocorrelation were accepted; model refinement was based on parsimony and the Akaike Information Criterion. Figure 4 shows the accurate prediction of arousal in a time series model based solely on intensity and autoregressive properties.

All the best time series models were nested within that for the Dvorak original intensity arousal response: where darousal was well modelled by lags 1–12 of dintensity (where 1 lag = 250 ms), with lags 1–5 of the autoregression (ar), and a moving average (ma) window of 19. No constant was needed. This model gave a correlation of forecast with observed data of .86 (Fig. 4 illustrates this). All of the models for the other series used the same dintensity lags, and at least lag 1 of the autoregression (ar), while none required the ma term, and in summary, beyond this their additional features are shown in Table 2. These models confirmed the substantial impact of intensity upon perceived arousal for all the pieces (as did impulse response function analysis, a technique which is discussed below).

**Table 2:** Piece Specific Features of ARIMAX models.

Model	Autoregressive components	Correlation between forecast and observed data
dtonalarousal	ar(2,3,4)	.77
datonalarousal	ar(2,3)	.69
dnoisearousal	ar(3,4,5)	.62
Dvorak inverted intensity piece, darousal	ar(2,3,4,5)	.61

All models shared the following ARIMAX terms: lags 1–12 of dintensity, and lag 1 of autoregression.

### Possible roles of musical expertise

We investigated two further aspects of the impact of intensity on perceived arousal, judged above both by controlled experimental manipulation (the Dvorak intensity inversion experiment) and by comparative response pattern analysis (across four very different musical entities which solely shared an intensity profile). First we considered the possible influence of musical expertise. For example, perhaps musicians, or even simply people familiar with a piece, might have learned responses to structural components other than intensity. More than 97.2% of participants indicated that they had some level of familiarity with both the Dvorak original and its inverted transform. Thus they were familiar at least with the genre it exemplifies, and so for this piece familiarity differences seem unlikely to have had an impact.

We investigated a possible role of musical expertise on the basis of participant OMSI scores. We first divided our participants into two equal-sized groups, split at the median OMSI. We assessed the relationship between intensity and arousal separately for the two groups. The mean time series of the arousal response for the two groups were virtually superimposable upon each other and upon the whole group grand average discussed above, both for the original Dvorak and for its intensity inverted transform. From this, the influence of intensity on the perception of arousal does not seem to depend on musical expertise.

The median split in our participant group was at an OMSI of 226. Given that the OMSI is a probability (×1000) that an individual would be judged by a group of musical experts as having a musical expertise beyond 5 on a scale from zero to 10, a ‘more musical’ group could be defined as one with OMSI values >500. Only seven of our participants were in this group (OMSI range 801–956), and so only brief comments about them are warranted, and the issue of expertise requires further investigation. First the average Dvorak arousal profile of the musicians was clearly different from the grand average, showing different levels (and a CV of 0.08), but the mirroring effect of the inversion was still apparent. The key parameters of the high OMSI group are shown in Table 3.

**Table 3:** Procrustes values (*d*) for the Dvorak extracts from the high OMSI group.

	Dvorak Inverted Arousal	Dvorak Original Intensity
Dvorak Original Arousal	0.0003	0.0003
Dvorak Inverted Intensity	0.0005	

The *d* values confirm that even for the high OMSI group, the arousal responses were similar to the intensity profiles engendering them, and to each other, as for the grand average group. Music-structural features beyond intensity may well influence these more musical participants, and their response lag structure is another possible distinctiveness. Time series analysis by ARIMAX, as above, showed that a significant influence of intensity remained in the best Dvorak original darousal model for the high OMSI group. Differencing gave stationarity, and the model was again nested within that of the whole participant group grand average described above, and contained lags 1–4, 7, 8, 10 and 12 of dintensity, and autoregressive lags 1, 3, and 4. It had a lower correlation of forecast with observed data (0.43) than that of the grand average model (0.86). Similarly, the best model for predicting the arousal response of the high OMSI group with the inverted Dvorak contained only lags 1, 6, 8, 10 and 12 of intensity, and autoregressive lags 1, 2, and 4: it had a correlation of forecast with observed data of 0.35. Thus intensity is still influential upon the arousal perceived by the high OMSI subgroup, though their performance is clearly different from the majority of our participants.

### Possible roles of loudness perception in the effect of acoustic intensity on perceived arousal

There is little literature relating perceived loudness of music (as opposed to computed intensity or computed ‘loudness’) to affective perceptions such as arousal. Loudness is defined as the perceptual counterpart of acoustic intensity, and computational models of loudness have intensity as the key quantitative influence [26], [27]. With few exceptions [28], [29] these models relate to constant sounds, or to short and simple if inconstant sounds (up to about 30 seconds). Thus we here assessed continuous perceptual loudness in relation to intensity, and considered its relation to perceived arousal. As part of a larger study, the loudness responses of 24 listeners (8 female, mean age 21 years) to the first 65 sec (only) of the Dvorak original and of the intensity-inverted version were determined. The temporal resolution of this study was lower (2 Hz) than used above, in part because loudness perception improves with increasing duration up to about 300 msec [30]. The loudness profiles were almost superimposable upon the corresponding intensity profiles (down-sampled to 2 Hz), both for the original and inverted versions of the Dvorak.

As expected, intensity seemed to strongly determine loudness perception. In other work we assess whether additional aspects of musical structure can perturb loudness perception, or conversely whether there is a largely ‘bottom up’ influence of intensity alone. Our evidence suggests the latter, and so here we considered whether this necessarily means that loudness is the mediator of the effect of acoustic intensity on arousal. Intensity is experienced through auditory and proprioceptive routes [31], even when music is played through headphones, so it might act directly on the perception of arousal in a piece, and/or via the influence of loudness. Intensity may also act more quickly on or in parallel with perceptions of arousal than on those of loudness; in either case, apparently direct impacts of intensity on arousal would occur, with lesser impact of loudness. Time series analysis can suggest which possibilities are pertinent for empirical investigation in the future.

We therefore investigated Granger causality relationships between arousal, intensity and loudness. VAR may be done with the conservative assumption that all variables potentially influence each other: they are ‘endogenous’ in statistical terms (like psychological dependent variables). When this is done with the Dvorak original and inversion series (standardised but undifferenced), intensity is Granger-causal on arousal (*p*<.000 and *p* = .020, respectively). However, loudness is also weakly Granger-causal on arousal in the original only (*p* = .048). Results were similar when the assessment was run on the stationarized first difference series. Thus VAR suggests that intensity acts directly on perception of the music's arousal level, with only secondary mediation by loudness perception.

**Figure 5:**
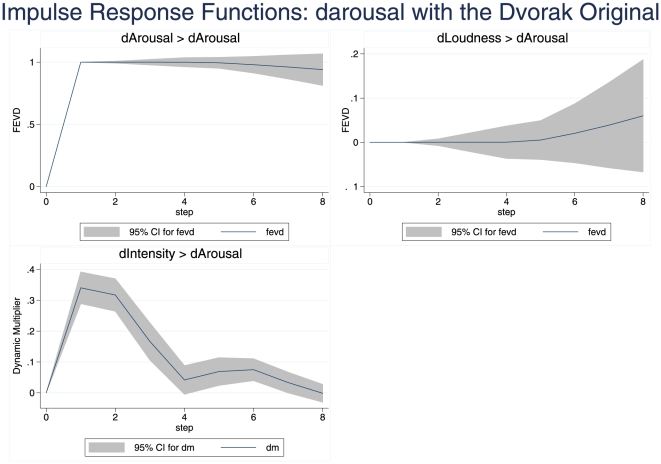
Impulse Response Functions for the dependence of darousal on dintensity and dloudness - Dvorak Original. Vector Autoregression of the stationarized darousal series for the original Dvorak melody (first 65 sec), with dintensity as an exogenous variable. Variables were standardised, and the figure shows the effect of a unit increase in each of the predictor variables, for 8 lags (each 0.5 sec) after the increase. dm = dynamic multiplier, the change in darousal due to one unit change in the exogenous variable. fevd = fractional error variance decomposition, the proportion of variance of darousal that might be explained by unit change in the predictor endogenous variable. The shaded areas reveal the 95% confidence limits of the responses (estimated by bootstrapping). In an autoregressive system it is to be expected that the response variable, darousal, will be a good predictor of itself, as shown. dintensity is also a significant influence, while dloudness is not; the overall model is highly significant (*p*<.0000).

This was investigated further by conducting a more realistic, less conservative, VARX in which intensity (lags 1–4) was taken as an exogenous (independent) variable (X) with loudness and arousal as endogenous variables. Assessment of the impulse response function reveals the statistical impact of unitary change in the endogenous variables on each other, and of the exogenous on the endogenous variables. With standardised but undifferenced variables, satisfactory models (i.e. with white noise residuals for the model of arousal, and a very close fit of predicted and data) could be obtained. Errors in the impulse response function are bootstrapped. Figure 5 shows that during the first 8 steps (lags) after any particular starting point in the arousal response, as expected the arousal at the starting point is an important predictor of itself. But more importantly, at step one a significant impulse effect of intensity is observed declining progressively thereafter. The impact of an exogenous variable is measured as a Dynamic Multiplier, the effect of a one unit change in an exogenous variable on the endogenous variables: so by step 1 a unit change in intensity creates 0.34 units of change in perceived arousal. The 95% confidence limits for this impulse do not breach zero until step 4, and hence it is highly significant. In contrast, the response to loudness changes is never significantly different from zero. Results for the corresponding VARX on stationarized (differenced) variables were closely similar. In the case of the intensity inverted Dvorak, again it was intensity that provided a statistically significant impulse on arousal (and it cumulated over more lags than with the original Dvorak), while loudness did not. The impulse response functions also confirm clearly the strong impact of intensity changes on perceived loudness changes.

## Discussion

The inversion of perceived arousal in response to the inversion of intensity in the Dvorak, with no other changes to the piece, demonstrates directly that intensity is a powerful influence on perceived arousal in this case. Furthermore, the shared arousal pattern of the original Dvorak, and the constructed tonal, atonal, and noise pieces confirms that intensity powerfully influences perceived arousal in a wide range of musical contexts. This does not exclude the possibility that other time-varying musical features (e.g. tempo [32]) are also powerful influences on arousal. It will be interesting to study how perceived arousal varies within pieces that have very limited intensity change, such as some ambient music, and what factors influence such perceived arousal.

Our ‘more musical’ participants also show an influence of intensity on perceptions of arousal, although their performance is different from that of the majority of participants. Such differences may also include their learned responses to musical structure. Perceptual loudness clearly mirrors acoustic intensity very closely, and hence does not show evidence of learned responses. But it is intensity rather than perceived loudness that seems to more directly influence perceived arousal. This requires further focused empirical investigation.

Thus our data build upon the existing knowledge of a correlation between the timing of intensity/computed loudness and perceived arousal [3], providing strong evidence that intensity profiles are a major causal factor upon continuously perceived arousal in music. Future work might explore whether changes in musical sound intensity are equally important in causing listeners to experience or feel arousal, i.e. induce an emotional response [7]. Arousal is a key dimension in many theories of emotional response to music [12], but it is only one, albeit important, component. Other aspects of musical affect and meaning are relatively poorly understood, and it is here that other musical features may be most important [11]. Future research can extend the findings and method outlined here, to experimentally manipulate, for example, the spectral profile of music to determine its role in shaping listener perceptions of the valence, or positive/negative emotions, of music through time.

## Supporting Information

Audio S1 Stimulus based on extract from tonal version of Dean's *Mutase* (2008), with superimposed intensity profile.
(MP3)

Audio S2 Stimulus based on extract from atonal version of Dean's *Mutase* (2008), with superimposed intensity profile.
(MP3)

Audio S3 Stimulus based on sound extract from *soundAffects* (2003) with superimposed intensity profile.
(MP3)
